# The first microbial environment of infants born by C-section: the operating room microbes

**DOI:** 10.1186/s40168-015-0126-1

**Published:** 2015-12-01

**Authors:** Hakdong Shin, Zhiheng Pei, Keith A. Martinez, Juana I. Rivera-Vinas, Keimari Mendez, Humberto Cavallin, Maria G. Dominguez-Bello

**Affiliations:** Division of Translational Medicine, New York University School of Medicine, 550 1st Avenue, BCD 690, New York, NY 10016 USA; Department of Veterans Affairs New York Harbor Healthcare System, New York, NY USA; Hospital Universitario, Medical Science Campus, University of Puerto Rico, Puerto Rico, USA; School of Architecture, University of Puerto Rico, Puerto Rico, USA

## Abstract

**Background:**

Newborns delivered by C-section acquire human skin microbes just after birth, but the sources remain unknown. We hypothesized that the operating room (OR) environment contains human skin bacteria that could be seeding C-section born infants.

**Results:**

To test this hypothesis, we sampled 11 sites in four operating rooms from three hospitals in two cities. Following a C-section procedure, we swabbed OR floors, walls, ventilation grids, armrests, and lamps. We sequenced the V4 region of the 16S *rRNA* gene of 44 samples using Illumina MiSeq platform. Sequences were analyzed using the QIIME pipeline. Only 68 % of the samples (30/44, >1000 sequences per site) yielded sufficient DNA reads to be analyzed. The bacterial content of OR dust corresponded to human skin bacteria, with dominance of *Staphylococcus* and *Corynebacterium*. Diversity of bacteria was the highest in the ventilation grids and walls but was also present on top of the surgery lamps. Beta diversity analyses showed OR dust bacterial content clustering first by city and then by hospital (*t* test using unweighted UniFrac distances, *p* < 0.05).

**Conclusions:**

We conclude that the dust from ORs, collected right after a C-section procedure, contains deposits of human skin bacteria. The OR microbiota is the first environment for C-section newborns, and OR microbes might be seeding the microbiome in these babies. Further studies are required to identify how this OR microbiome exposure contributes to the seeding of the neonatal microbiome. The results might be relevant to infant health, if the current increase in risk of immune and metabolic diseases in industrialized societies is related to lack of natural exposure to the vaginal microbiome during labor and birth.

**Electronic supplementary material:**

The online version of this article (doi:10.1186/s40168-015-0126-1) contains supplementary material, which is available to authorized users.

## Background

The mother is an important source of the first microbiome for infants [1]. Regardless of the possible in utero exposure to bacterial components [2, 3], mammals are exposed during labor to a dense vaginal inoculum that is later subjected to the selective pressure of milk components with prebiotic effects. These exposures, which are likely adaptive, are altered in mammalian infants born by C-section who lack vaginal exposure during birth.

We have previously shown that C-section born infants acquire skin-like bacteria (*Staphylococcus*, *Corynebacterium*, and *Propionibacterium*) at birth [4]. The source of this human skin microbiota that first seeds C-section born infants remains unknown. Humans shed up to 37 million bacterial genomes into the environment per hour [5, 6]. Operating rooms (ORs) are occupied by humans, lack natural ventilation, and, regardless of the efficacy of cleaning, are expected to be highly enriched with human skin bacteria [7–10]. In this work, we characterized bacterial contents in dust collected from ORs.

## Methods

We sampled several sites in ORs immediately following C-section procedures and identified bacterial contents in dust collected with sterile swabs, using 16S *rRNA* gene sequencing. In addition, we used standard culturing methods to determine the presence of live bacteria in OR dust deposits.

### Sample collection

Environmental samples were obtained from 11 sites in each OR (Additional file 1: Figure S1) by rubbing sterile swabs pre-moistened with 0.15 M NaCl solution with 0.1 % TWEEN 20. Whole surfaces of each site were swabbed except on walls and floors (swabbed from one square meter area). Samples (*n* = 44, Additional file 2: Table S1) were collected from four ORs from three hospitals in two cities (New York, NY and San Juan, PR). Negative control swabs (*n* = 3) were also included. All swabs were immediately frozen at −80 °C, until DNA extraction.

### DNA extraction and sequencing

Total DNA was extracted using the MoBio (CA, USA) PowerSoil®-htp 96 Well Soil DNA Isolation plates according to the manufacturer’s procedure. The V4 region of the 16S *rRNA* gene was amplified by PCR using barcoded primers and was sequenced using the paired-end technique (Illumina Miseq platform), as previously described [11].

### Data analysis

The 16S *rRNA* sequence analyses were conducted using the QIIME suite of software tools (v1.8) [12]. The operational taxanomic units (OTUs) were picked from filtered sequence reads (Phred ≥ Q20) with an open-reference OTU picking method based on 97 % identity with the Greengenes database (v13_8). Chimeric sequences were discarded using the ChimeraSlayer method [13]. All communities were rarefied to 3194 reads per sample to calculate bacterial diversity. For comparison of beta diversity, the unweighted and weighted UniFrac distances were calculated [14]. To test for significance of the inter- and intra-group distance differences, non-parametric *t* tests were used with 999 permutations. For multivariate analysis of variance, PERMANOVA (permutational ANOVA) was used with 999 permutations [15]. In multiple comparisons, Bonferroni-corrected *p* values were calculated. Linear discriminant analysis effect size (LEfSe) [16] was used to detect unique biomarkers (LDA score >3.0) in relative abundance of bacterial taxonomy.

To compare OR samples with the Human Microbiome Project (HMP) database [17], the HMP dataset of 16S *rRNA* (V3-5 region) sequences was downloaded from the NIH HMP website (hmpdacc.org). BioPerl (Bioperl.org) was used to trim this dataset to have only V4 region of 16S *rRNA*. QIIME suite (v1.8) was used to pick OTUs from the HMP dataset with OR samples using the closed-reference method. Then, all communities were rarefied to 1000 sequences per sample to calculate bacterial beta diversity.

To determine the possibility that OR dusts are a microbial source for the infant microbiota, we predicted microbial sources in infant skin sites (1–7 days after birth; forehead, volar, and foot) using the SourceTracker method, as previously described [18], to analyze samples available from our infant development project (IRBs from the University of Puerto Rico A9710112 and 1011–107: seven infants born vaginally and ten born by C section; 16S *rRNA* V4 sequences available at the EBI-European Nucleotide Archive: ERP012216).

### Microscope observation

For microscopic examination, a swabbed dust sample was mixed with twofold diluted bovine serum (Thermo Scientific, MA, USA) and smeared on an adhesive microscope slide (Mercedes Medical, FL, USA). The air-dried smear was stained with hematoxylin and eosin stain. As a positive control, scrubbed human skin flakes were prepared with same procedure.

An aliquot of the swab sample was also fixed in 10 % formalin overnight, washed twice in Dulbecco’s phosphate-buffered saline (PBS; Life Technologies Grand Island, NY, USA) and re-suspended in a minimal amount of PBS. Cell debris was captured using the plasma-thrombin clotting technique [19], processed using standard histological tissue processing methods, and subsequently embedded in paraffin wax. The embedded sample was sectioned at 4 μm with representative sections stained with hematoxylin and eosin. Immunohistochemistry was performed on formalin-fixed paraffin-embedded 4-μm-thick sections using mouse anti-human Pan-cytokeratin (Molecular Probes Cat# 985542A, RRID: AB_2335731) clone AE1/AE3. Immunohistochemistry was performed on a Ventana Discovery platform using Ventana’s reagents and detection systems (Ventana Medical Systems, AZ, USA). Slides were deparaffinized and antigens retrieved in Ventana Cell Conditioner 1 (Tris-Borate-EDTA, pH 8.5) for 28 min (mild setting). Endogenous peroxidase activity was blocked with 3 % hydrogen peroxide for 4 min. Anti-pan-keratin was diluted 1:100 in Dulbecco’s PBS and incubated 30 min. Primary antibody was detected by the application of a biotinylated goat anti-mouse for 8 min, followed by the application of streptavidin-horseradish peroxidase for 8 min. The chromogen, 3,3’-diaminobenzidine/hydrogen peroxide mix was applied for 8 min and then enhanced with copper sulfate for 4 min. Slides were then counterstained with hematoxylin, dehydrated, and mounted with permanent media.

### Availability of supporting data

The raw sequences supporting the results of this article are available in the European Nucleotide Archive repository as PRJEB11484 (http://www.ebi.ac.uk/ena/data/view/PRJEB11484). Supplementary information is included with the article and available on the Microbiome website.

## Results

Of the 44 OR samples collected, 68 % (30/44, >1000 sequences per site) had a sufficient number of DNA sequences to be analyzed (Additional file 2: Table S1). A total of 367,086 sequences (paired-end, Phred ≥ Q20) were obtained from these samples, and the average sequence number per sample was 12,236 ± 5171. These sequences were binned into 3638 types of OTU (Additional file 3: Table S2). And, Blank swabs (*n* = 3) had 53 sequences, consisting 15 genus-level taxa (<6 sequences per OTU, Additional file 4: Table S3).

**Fig. 1:**
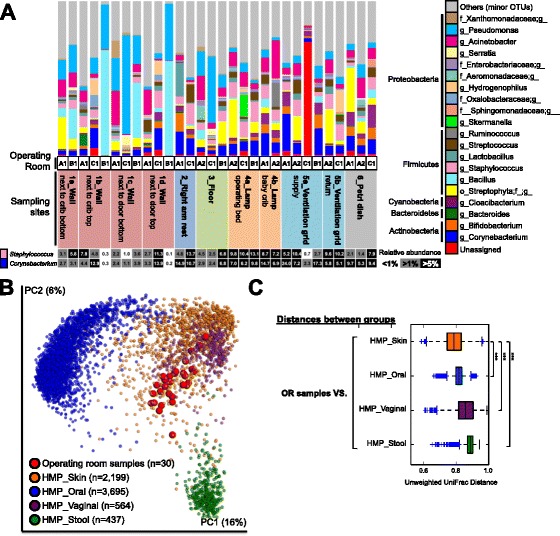
Bacterial diversity in operating rooms. **a** Bacterial taxa plot at the genus-level. Major phylotypes (>1 % of relative abundance at least one sample) were indicated by each color. The relative abundances of *Staphylococcus* and *Corynebacterium* were represented by heat map (*Bottom*). **b** PCoA plot of bacterial communities of OR samples with HMP database. Unweighted UniFrac distances were used to evaluate diversities between samples. **c** Box plots of inter-group distances of bacterial communities between OR samples and HMP database. ***Non-parametric *p* < 0.001

Notably, all analyzed samples (*n* = 30) contained human skin bacteria with dominance of *Staphylococcus* and *Corynebacterium* (Fig. 1a). While there were no correlations of bacterial composition by sampling sites, lamps (on the operating bed and baby crib) showed higher relative abundances of *Staphylococcus* and *Corynebacterium* than other sites (Kruskal–Wallis test, *p* < 0.05; Fig. 1a). Ventilation grids for air return contained the highest bacterial diversity, followed by wall samples nearest the floor, floors, and the top of lamps over the operating bed with non-statistical tendency (Additional file 5: Figure S2). Moreover, live bacteria (*Staphylococcus*) were grown on blood agar plates, using standard plating methods, from swabs of the tops of operating room lamps (Additional file 6: Table S4).

In addition, the microbiota of OR samples was more similar to human skin microbiota (HMP database; non-parametric *t* test using unweighted UniFrac distance, *p* < 0.001; Fig. 1b, c) compared to other body sites (oral, feces, vaginal). Consistently, we detected human skin flake-like cells in OR samples using microscopic observation with H/E and Pan-keratin staining (Additional file 7: Figure S3), suggesting dust from ORs contains deposits of human skin flakes that could be a carrier of live human skin bacteria.

Based on our SourceTracker analyses, the skin microbiota of infants born by C-section has a high proportion of bacteria from the OR compared to vaginally born infants, whose skin microbiota has a low proportion of OR bacteria and a high proportion of maternal vaginal bacteria (volar; *p* < 0.05, *t* test, Additional file 8: Figure S4).

Bacterial beta diversity on principal coordinates analysis (PCoA) plot showed that microbes clustered separately according to hospital (Additional file 9: Figure S5) in addition to clustering by city (non-parametric *t* test using unweighted UniFrac distances, *p* < 0.05; PERMANOVA, *p* < 0.1). OR “A2” showed more convergence in bacterial community structure than other ORs (non-parametric *t* test using unweighted UniFrac distances, *p* < 0.005; Additional file 9: Figure S5C). Weighted UniFrac distance matrix results also supported these results (Additional file 10: Figure S6).

There were no significant differences in alpha diversity between hospitals (Additional file 11: Figure S7), but environmental taxa differentiating hospitals included *Bacteroides*, *Shuttleworthia*, *Acinetobacter*, *Ruminococcus*, *Bacillus*, *Hyphomicrobium*, *Helcococcus*, and *Hydrogenophilus* (by abundance; Additional file 9: Figure S5E and Additional file 12: Figure S8).

While there was no significant segregation between bacterial communities by sampling site, the microbiota from ORs showed a non-significant tendency toward clustering between the top or bottom of the walls and floors (Additional file 13: Figure S9).

## Discussion and conclusions

While modern operating rooms are expected to have aseptic environments, several studies have already reported microbial presence in ORs using culture-dependent methods, pulse-field gel electrophoresis, fluorescent particle counting, and adenosine triphosphate (ATP) testing [10, 20, 21]. In the present study, we used 16S *rRNA* gene sequencing to show that OR dust, collected right after a C-section procedure, contains bacteria similar to human skin microbiota. Previous studies using culture-dependent methods also showed that over 85 % of air samples from ORs had skin-like bacteria which were mostly coagulase-negative staphylococci and *Corynebacterium* [10]. These airborne skin-bacteria could be from individuals present during C-section but could also be shed by cleaning personnel between operations.

In our study, ~30 % of samples failed to yield sufficient DNA sequences to be analyzed. While there are no published data on the microbiota in operating rooms using 16S *rRNA* gene sequencing, very few bacteria (average 3.3–3.5 CFU/10 cm^2^) were detected in ORs after regular decontamination using standard culturing methods [22, 23], consistent with the low sequence numbers in our study. However, there was variation between two ORs from the same hospital, with similar wall materials and hygiene procedures (e.g., A1 walls yielded higher bacterial sequences than A2 walls). Sampling and hygiene procedure timings may have had an effect on the detected sequence numbers. Further studies are needed to elucidate the dynamics of indoor environmental conditions like the ongoing Hospital Microbiome Project [24] and associated variations in the microbial content of hospital environments.

The top of OR lamps, which are hard to reach and clean, have deposits of dust containing live skin bacteria, which when moved by the surgeon, might create a bacterial plume that sheds on the newborn. Petri dishes placed on the floors collected particles with similar relative abundances to skin bacteria, suggesting that ORs have airborne skin bacteria that accumulate on surfaces. Patient warming systems in general surgery rooms generate air convection currents that circulate resident air from the floor up to the ceiling [25], which may also help circulate airborne bacteria in ways independent from transfer by direct contact [26].

In addition, we found that the microbiota of OR samples was more similar to human skin microbiota than oral microbiota and that OR dust contains deposits of human skin flakes. These results reveal that while the use of surgical masks has limited effectiveness at curtailing oral microbial shedding [27], skin flakes from individuals present during C-section and/or from cleaning personnel between operations could be a more influential factor contributing to the structure of OR microbiota.

Our SourceTracker analysis results suggest that the OR microbes could play a role in seeding infants born by C-section. C-section born infants, in particular, may be solely receiving this inoculum, while vaginally born infants have exposure to vaginal bacteria. The results of these further studies could be relevant to the possible effects on the priming of the immune system by skin bacteria from environmental sources as the primordial inoculum seeding the infant microbiome. This might be relevant to the increased risk of immune diseases observed in C-section born infants [28, 29].

## Additional files

Additional file 1: Figure S1. Schematic diagram of sampling sites in operating rooms. Environmental samples were obtained from 11 sites in 4 operating rooms from three hospitals in two cities. (PDF 355 kb) Additional file 2: Table S1. The distribution of sequences in collected samples. Only OR samples having more than 1,000 sequences were used for further analyses. (PDF 43 kb) Additional file 3: Table S2. Sequecing information for OR samples. A total of 353,085 sequences were binned into 3,638 different OTUs with an open-reference OTU picking method based on 97% identity, with the Greengenes database (v13_8). (PDF 303 kb) Additional file 4: Table S3. Bacterial OTUs detected in blank samples. Each OTU ID was assigned with an open-reference OTU picking method based on 97% identity with the Greengenes database (v13_8). (PDF 43 kb) Additional file 5: Figure S2. Box plots of bacterial alpha diversity by sampling site using PD whole tree matrix (Left) and number of observed species (Right). Each color represent a sampling site. (PDF 67 kb) Additional file 6: Table S4. BLASTN results of 16S *rRNA* genes from bacterial cultures from OR dust.Sequences were blast aginst NCBI database. (PDF 48 kb) Additional file 7: Figure S3. Microscopic observation of positive control (scrubbed human skin flakes, left), OR sample (middle), and negative control (right) with H/E staining and formalin fixation with H/E staining, formalin fixation and H/E staining (serum fixation). (PDF 9,315 kb) Additional file 8: Figure S4. Source proportions for infants skin sites (foot, forehead, and volar) predicted using SourceTracker. The average contributions of human (all the mother’s sites) and operating room sources to the infant (1–7 days after birth) skin bacterial communities were predicted by SourceTracker. (PDF 109 kb) Additional file 9: Figure S5. Bacterial diversity of operating rooms by location. A. PCoA plot of bacterial communities of OR samples. Unweighted UniFrac distances were used to evaluate diversities between samples. B. Box plots of inter-group distances of bacterial communities between ORs. ***p* < 0.05; ****p* < 0.01. C. Box plot of intra-group distances of bacterial communities. ****p* < 0.01. D. PERMANOVA *p* values of inter-group. E. Unique biomarker bacteria in each OR. LDA Effect Size (>3.0-fold) was used to detect unique biomarkers. (PDF 158 kb) Additional file 10: Figure S6. Beta diversity in OR samples using weighted UniFrac distances. A. PCoA plot of bacterial communities of OR samples. B. Box plots of inter-group distances of bacterial communities between ORs. ***p* < 0.05; ****p* < 0.01. C. Box plot of intra-group distances of bacterial communities. ***p* < 0.05. D. PERMANOVA *p* values of inter-group. (PDF 128 kb) Additional file 11: Figure S7. Rarefaction plots of OR microbiota by locations using PD whole tree matrix (Left) and number of observed species (Right). All communities were rarefied at 3,194 reads. (PDF 100 kb) Additional file 12: Figure S8. Bacterial taxa plot at the genus-level by OR locations. Major phylotypes (>1 % of relative abundance at least one sample) is indicated by different colors. (PDF 223 kb) Additional file 13: Figure S9. PCoA plot of bacterial communities in each OR. In upper panels, unweighted UniFrac distances were used to evaluate diversities between samples. In bottom panels, weighted UniFrac distances were used to evaluate diversities between samples. (PDF 262 kb)

## Abbreviations

HMP: Human Microbiome Project
OR: operating room
OTU: operational taxonomic unit
PCoA: principal coordinates analysis
