# Erratum to: The first microbial environment of infants born by C-section: the operating room microbes

**DOI:** 10.1186/s40168-016-0148-3

**Published:** 2016-01-21

**Authors:** Hakdong Shin, Zhiheng Pei, Keith A. Martinez, Juana I. Rivera-Vinas, Keimari Mendez, Humberto Cavallin, Maria G. Dominguez-Bello

**Affiliations:** Division of Translational Medicine, New York University School of Medicine, 550 1st Avenue, BCD 690, New York, NY 10016 USA; Department of Veterans Affairs New York Harbor Healthcare System, New York, NY USA; Hospital Universitario, Medical Science Campus, University of Puerto Rico, Puerto Rico, USA; School of Architecture, University of Puerto Rico, Puerto Rico, USA

After publication of this article [1], the authors noticed one of the collaborator’s names had been misspelled in the ‘Acknowledgements’ section. “Dr. Tsei Ming” is incorrect and should be “Dr. Ming C. Tsai”. The correct version of the ‘Acknowledgements’ section is included below and has been updated in the original article [1].
