# Supplementary figures and images for: The first microbial environment of infants born by C-section: the operating room microbes
Source: Microbiome. 2015 Dec 1;3:59. doi: 10.1186/s40168-015-0126-1 (PMC4665759; doi:10.1186/s40168-015-0126-1)

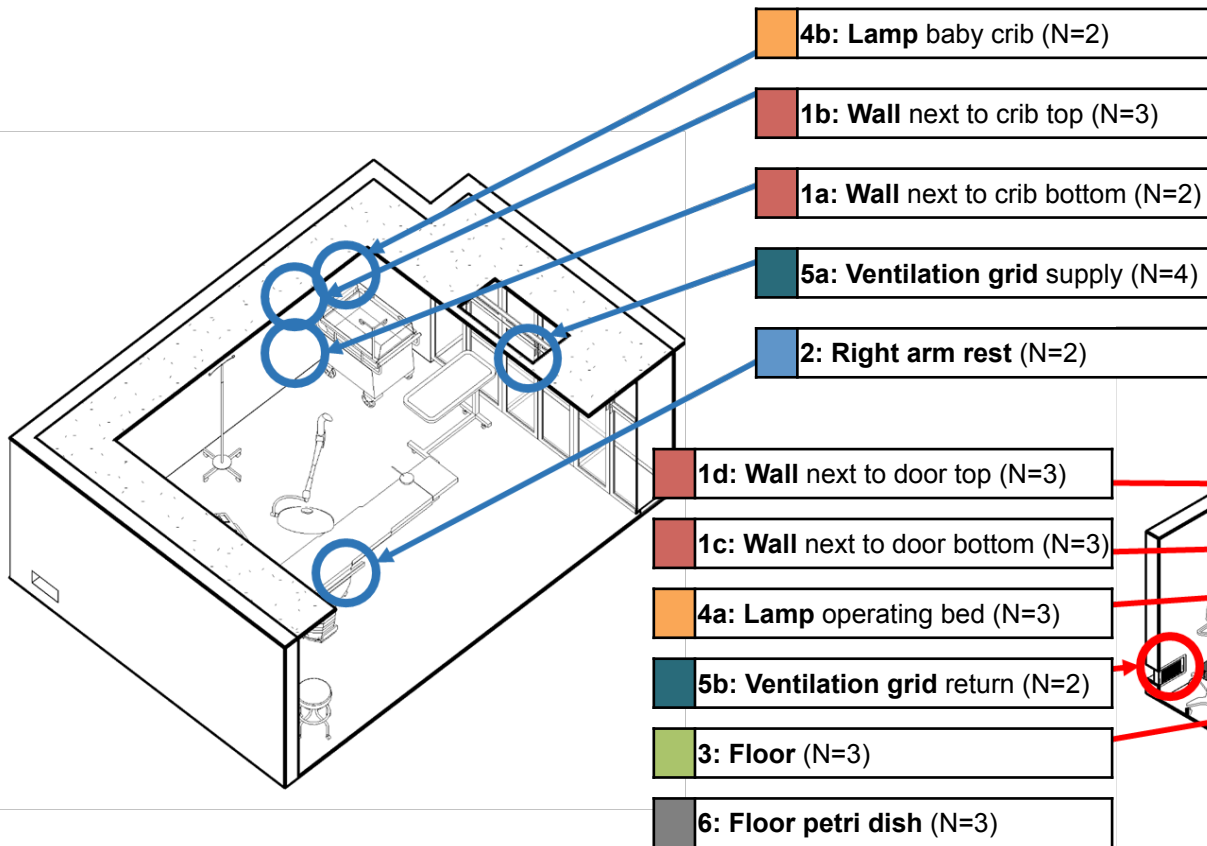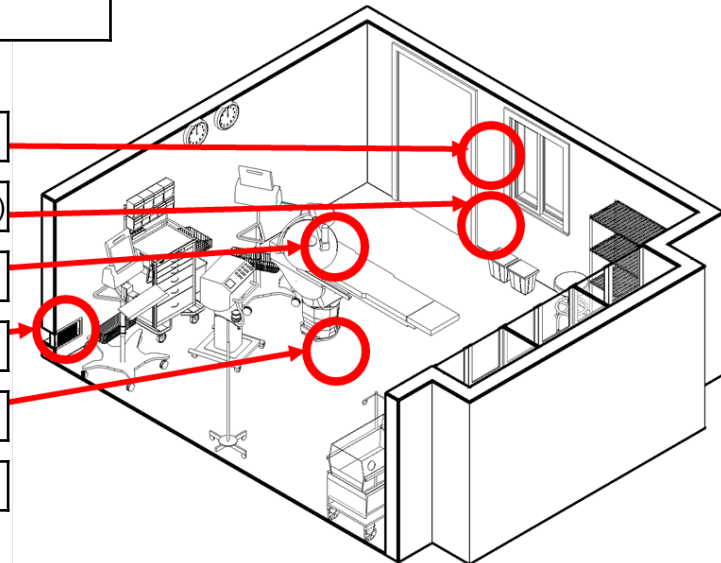

Supplement: Additional file 1: Figure S1. — Schematic diagram of sampling sites in operating rooms. Environmental samples were obtained from 11 sites in 4 operating rooms from three hospitals in two cities. (PDF 355 kb) [file 40168_2015_126_MOESM1_ESM.pdf]

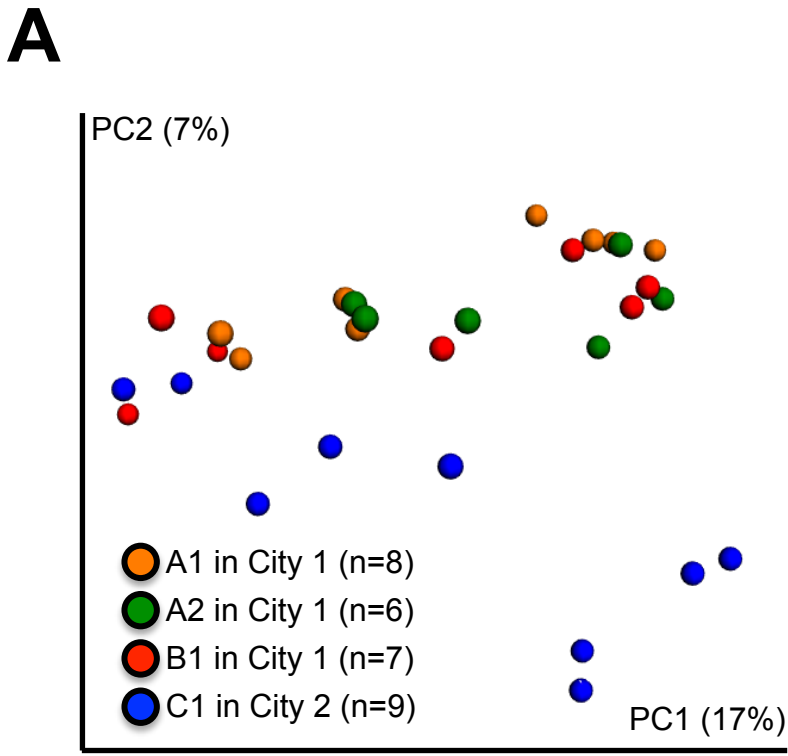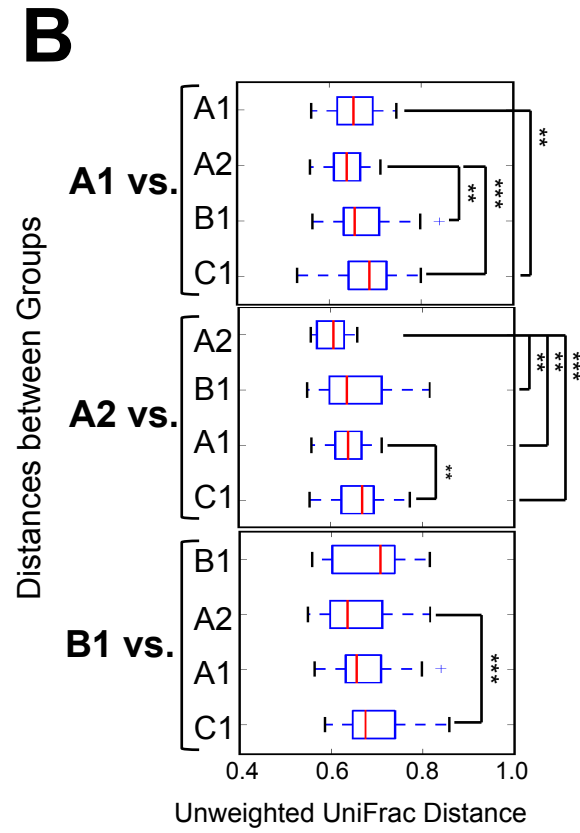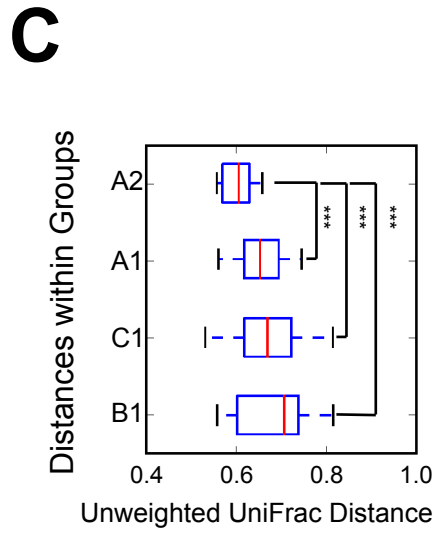

### D

**PERMANOVA**  
(p-value)

|    | A1    | A2    | C1    |
|----|-------|-------|-------|
| A2 | 0.121 | -     | -     |
| C1 | 0.062 | 0.053 | -     |
| B1 | 0.333 | 0.181 | 0.138 |

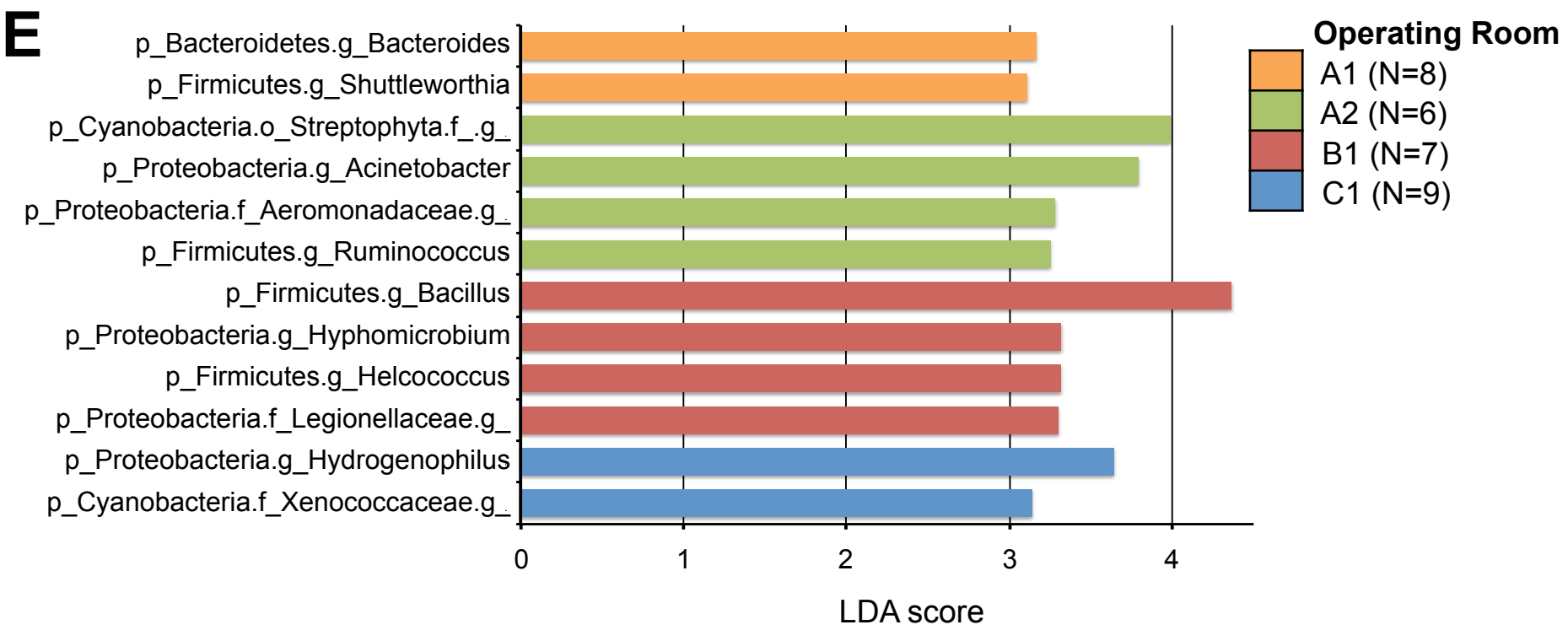

Supplement: Additional file 9: Figure S5. — Bacterial diversity of operating rooms by location. A. PCoA plot of bacterial communities of OR samples. Unweighted UniFrac distances were used to evaluate diversities between samples. B. Box plots of inter-group distances of bacterial communities between ORs. **p < 0.05; ***p < 0.01. C. Box plot of intra-group distances of bacterial communities. ***p < 0.01. D. PERMANOVA p values of inter-group. E. Unique biomarker bacteria in each OR. LDA Effect Size (>3.0-fold) was used to detect unique biomarkers. (PDF 158 kb) [file 40168_2015_126_MOESM9_ESM.pdf]

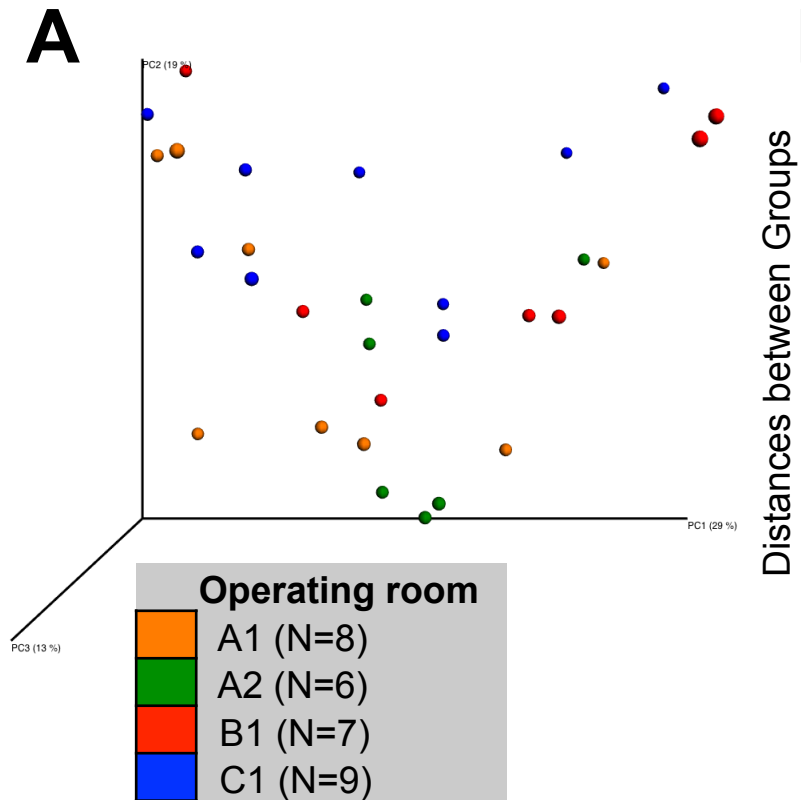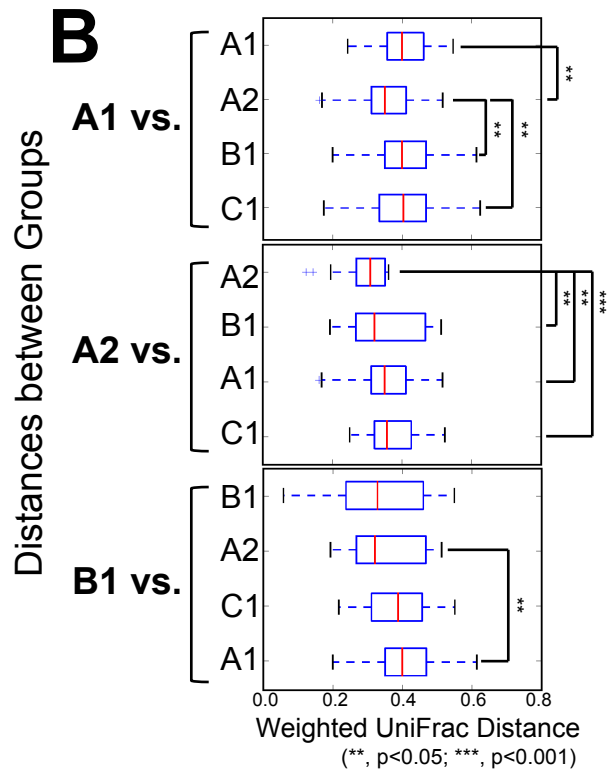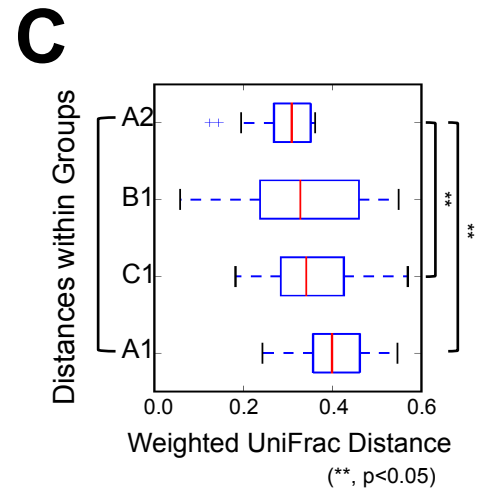

**D**

**PERMANOVA**  
(p-value)

|    | A1    | A2    | C1    |
|----|-------|-------|-------|
| A2 | 0.36  | -     | -     |
| C1 | 0.059 | 0.022 | -     |
| B1 | 0.12  | 0.071 | 0.082 |

Supplement: Additional file 10: Figure S6. — Beta diversity in OR samples using weighted UniFrac distances. A. PCoA plot of bacterial communities of OR samples. B. Box plots of inter-group distances of bacterial communities between ORs. **p < 0.05; ***p < 0.01. C. Box plot of intra-group distances of bacterial communities. **p < 0.05. D. PERMANOVA p values of inter-group. (PDF 128 kb) [file 40168_2015_126_MOESM10_ESM.pdf]

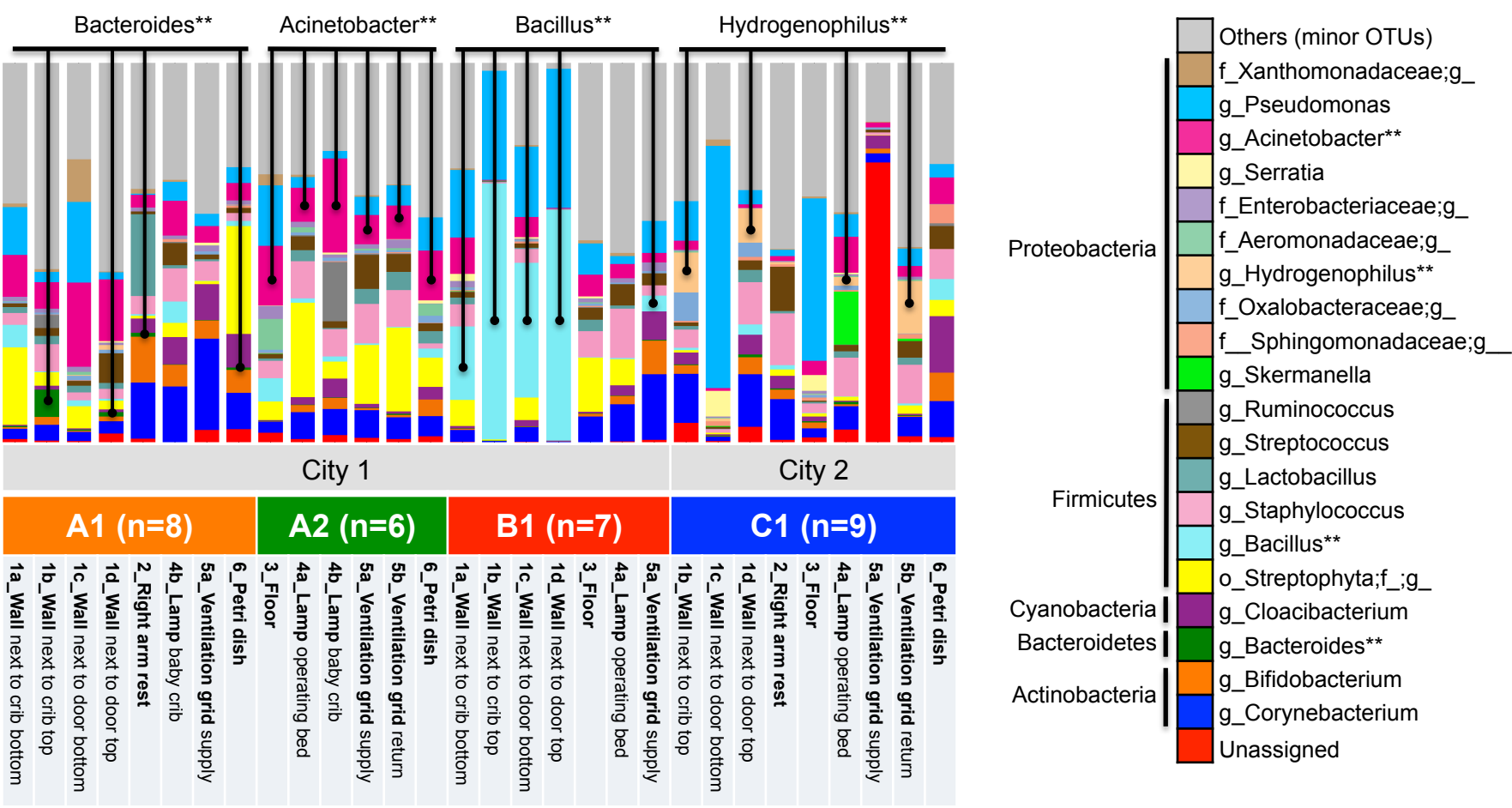

Supplement: Additional file 12: Figure S8. — Bacterial taxa plot at the genus-level by OR locations. Major phylotypes (>1 % of relative abundance at least one sample) is indicated by different colors. (PDF 223 kb) [file 40168_2015_126_MOESM12_ESM.pdf]
