# Supplementary material for: The first microbial environment of infants born by C-section: the operating room microbes
Source: Microbiome. 2015 Dec 1;3:59. doi: 10.1186/s40168-015-0126-1 (PMC4665759; doi:10.1186/s40168-015-0126-1)
Supplement: Additional file 2: Table S1. — The distribution of sequences in collected samples. Only OR samples having more than 1,000 sequences were used for further analyses. (PDF 43 kb) [file 40168_2015_126_MOESM2_ESM.pdf]

**Table S1. Distribution of sequences in collected samples**

| Sampling sites | | Operating room | | | |
|---|---|---|---|---|---|
| | | A1 | A2 | B1 | C1 |
| WALL | 1a Wall next to floor | 15,974 | 34* | 26* | 14,381 |
| | 1b Wall next to top crib | 12,649 | 39* | 4,410 | 15,923 |
| | 1c Wall next to bottom door | 13,464 | 199* | 17,727 | 14,531 |
| | 1d Wall next to top door | 4,785 | 16* | 8,441 | 18,571 |
| BED | 2 Right arm rest | 3,373 | 16* | 9,977 | 32* |
| FLOOR | 3 Floor | 18* | 22,272 | 18,499 | 14,898 |
| LAMP | 4a Lamp operating bed | 119* | 12,644 | 17,388 | 17,923 |
| | 4b Lamp baby crib | 7,369 | 13,075 | 19* | 39* |
| VENTILATION | 5a Ventilation grid supply | 9,094 | 18,282 | 6,676 | 7,097 |
| | 5b Ventilation grid return | 523* | 16,726 | 4,699 | 18* |
| PETRI DISH | 6 Petri dish | 9,681 | 12,076 | 4,481 | 17* |

*, Samples lacking enough number of DNA sequences
