# Supplementary material for: The first microbial environment of infants born by C-section: the operating room microbes
Source: Microbiome. 2015 Dec 1;3:59. doi: 10.1186/s40168-015-0126-1 (PMC4665759; doi:10.1186/s40168-015-0126-1)
Supplement: Additional file 3: Table S2. — Sequecing information for OR samples. A total of 353,085 sequences were binned into 3,638 different OTUs with an open-reference OTU picking method based on 97% identity, with the Greengenes database (v13_8). (PDF 303 kb) [file 40168_2015_126_MOESM3_ESM.pdf]

Table S2. Summary of analyzed sequence information

Characteristics	Operating room				Total
	A1	A2	B1	C1	
No. of samples	8	6	7	9	30
Total no. of Seqs	76389	95,075	103,324	92,298	367,086
Mean no. of Seqs (± Std dev)	9,549 ± 4,766	15,846 ± 5,669	14,761 ± 3,655	10,255 ± 3,481	12,236 ± 5,171
Total no. of Seqs yielding OTUs	73,388	91,649	98,103	89,945	353,085 (3.8% loss)
Mean no. of Seqs yielding OTUs (± Std dev)	9,174 ± 3,896	15,275 ± 3,567	14,015 ± 3,396	9,994 ± 5,548	11,770 ± 4,989
No. of observed OTU types	1,752	1,711	1,459	1,597	3,638
