# Supplementary material for: The first microbial environment of infants born by C-section: the operating room microbes
Source: Microbiome. 2015 Dec 1;3:59. doi: 10.1186/s40168-015-0126-1 (PMC4665759; doi:10.1186/s40168-015-0126-1)
Supplement: Additional file 4: Table S3. — Bacterial OTUs detected in blank samples. Each OTU ID was assigned with an open-reference OTU picking method based on 97% identity with the Greengenes database (v13_8). (PDF 43 kb) [file 40168_2015_126_MOESM4_ESM.pdf]

Table S3. Detected OTUs in blank swabs

	Detected Number				
OTU ID	NC_A1	NC_A2	NC_B1	Taxa_phylum	Taxa_genus
72820	2	0	0	p__Actinobacteria	g__Bifidobacterium
4381553	5	0	0	p__Bacteroidetes	g__Bacteroides
4439360	1	0	0	p__Bacteroidetes	g__Bacteroides
175485	1	0	0	p__Bacteroidetes	g__Bacteroides
3600504	0	5	0	p__Bacteroidetes	g__Bacteroides
3426658	0	1	0	p__Bacteroidetes	g__Bacteroides
3563235	0	1	0	p__Bacteroidetes	g__Bacteroides
577170	0	1	0	p__Bacteroidetes	g__Bacteroides
4256470	0	4	0	p__Bacteroidetes	g__Bacteroides
4154872	0	0	1	p__Bacteroidetes	g__Cloacibacterium
182643	0	1	0	p__Firmicutes	f__Clostridiaceae; g__
185607	2	0	0	p__Firmicutes	f__Lachnospiraceae; g__
289734	0	1	0	p__Firmicutes	f__Lachnospiraceae; g__
4457427	2	0	0	p__Firmicutes	g__Lachnospira
316732	1	2	0	p__Firmicutes	g__Lachnospira
2407149	0	1	0	p__Firmicutes	g__Lachnospira
3926480	2	0	0	p__Firmicutes	g__Roseburia
313593	1	0	0	p__Firmicutes	g__Roseburia
265871	2	1	0	p__Firmicutes	f__Ruminococcaceae; g__
191792	1	0	0	p__Firmicutes	f__Ruminococcaceae; g__
4439469	0	2	0	p__Firmicutes	f__Ruminococcaceae; g__
199145	1	3	0	p__Firmicutes	g__Faecalibacterium
1504042	0	1	0	p__Firmicutes	g__Oscillospira
146554	1	0	0	p__Firmicutes	g__Ruminococcus
16054	0	0	1	p__Firmicutes	g__Ruminococcus
4390365	0	1	0	p__Firmicutes	f__Erysipelotrichaceae;g__
4449236	1	0	0	p__Proteobacteria	g__Sutterella
274365	0	0	1	p__Proteobacteria	f__Enterobacteriaceae;g__
668514	0	1	0	p__Proteobacteria	f__Enterobacteriaceae;g__
584176	0	0	1	p__Proteobacteria	g__Acinetobacter
