# Supplementary material for: The first microbial environment of infants born by C-section: the operating room microbes
Source: Microbiome. 2015 Dec 1;3:59. doi: 10.1186/s40168-015-0126-1 (PMC4665759; doi:10.1186/s40168-015-0126-1)

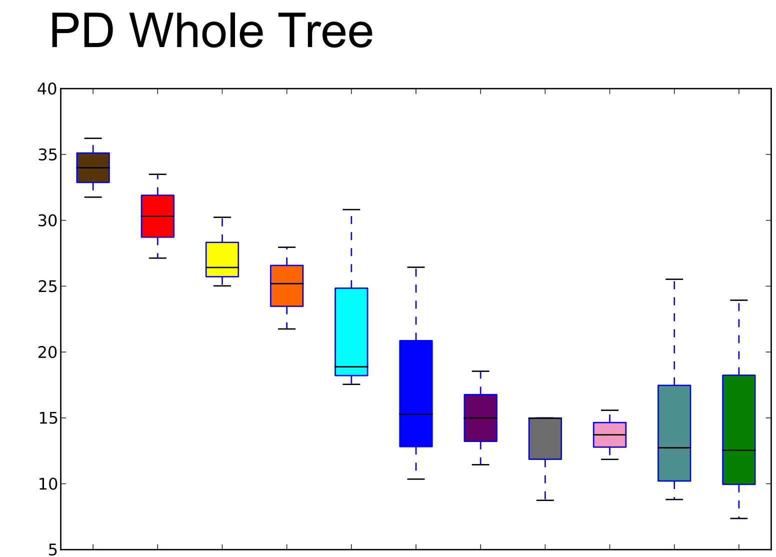

**Mean**	34.0	30.3	27.2	25.0	22.4	17.4	15.0	12.9	13.7	14.9	14.6
**Std**	2.2	3.2	2.2	2.5	6.0	6.7	3.5	2.9	1.9	6.5	6.9

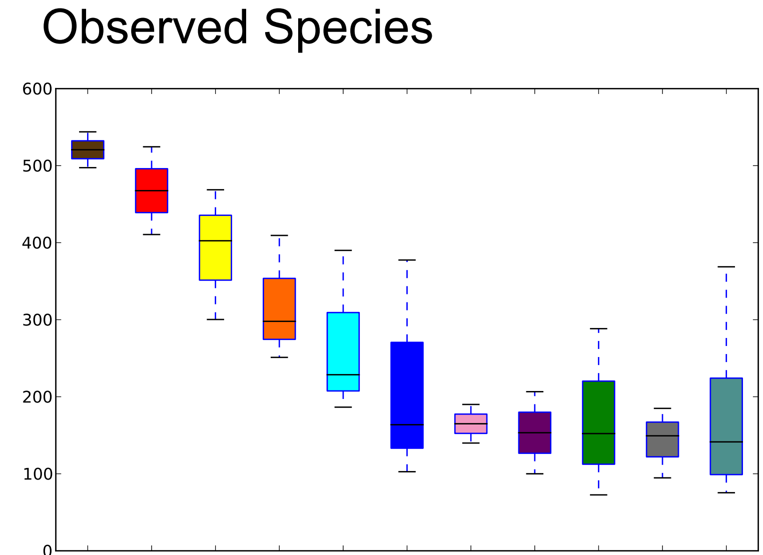

**Mean**	520.7	467.6	390.5	319.5	268.4	214.6	165.0	153.3	171.1	143.0	181.7
**Std**	23.3	57.0	69.3	66.4	87.7	117.8	25.1	53.3	89.1	37.1	113.9

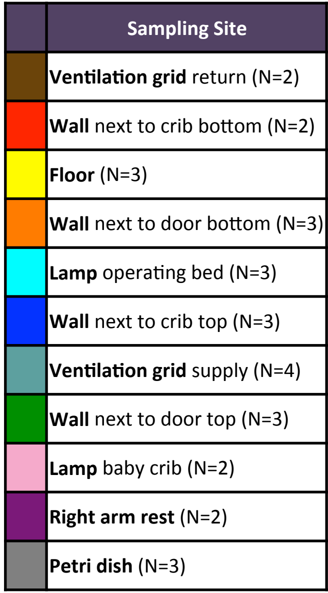

Supplement: Additional file 5: Figure S2. — Box plots of bacterial alpha diversity by sampling site using PD whole tree matrix (Left) and number of observed species (Right). Each color represent a sampling site. (PDF 67 kb) [file 40168_2015_126_MOESM5_ESM.pdf]
