# Supplementary material for: The first microbial environment of infants born by C-section: the operating room microbes
Source: Microbiome. 2015 Dec 1;3:59. doi: 10.1186/s40168-015-0126-1 (PMC4665759; doi:10.1186/s40168-015-0126-1)
Supplement: Additional file 6: Table S4. — BLASTN results of 16S rRNA genes from bacterial cultures from OR dust.Sequences were blast aginst NCBI database. (PDF 48 kb) [file 40168_2015_126_MOESM6_ESM.pdf]

Table S4. BLASTN results of 16S rRNA genes from live bacteria in OR dust

**Isolates**	**Query length (bp)**	**BLASTN best match (16S rRNA sequences)**	**Identities**	**Sequence ID**
OR_Isolate_5	644	*Staphylococcus petrasii* strain CCM 8418	98%	NR_118450
OR_Isolate_6	887	*Staphylococcus epidermidis* RP62A strain RP62A	97%	NR_074995
OR_Isolate_7	779	*Staphylococcus hominis* subsp. novobiosepticus strain GTC 1228	97%	NR_041323
OR_Isolate_8	576	*Staphylococcus petrasii* strain CCM 8418	98%	NR_118450
OR_Isolate_10	902	*Staphylococcus hominis* subsp. novobiosepticus strain GTC 1228	98%	NR_041323
OR_Isolate_11	966	*Staphylococcus hominis* subsp. novobiosepticus strain GTC 1228	99%	NR_041323
OR_Isolate_13	838	*Staphylococcus epidermidis* RP62A strain RP62A	98%	NR_074995
OR_Isolate_14	781	*Staphylococcus hominis* subsp. novobiosepticus strain GTC 1228	99%	NR_041323
OR_Isolate_19	937	*Staphylococcus epidermidis* RP62A strain RP62A	97%	NR_074995
