# Supplementary material for: The first microbial environment of infants born by C-section: the operating room microbes
Source: Microbiome. 2015 Dec 1;3:59. doi: 10.1186/s40168-015-0126-1 (PMC4665759; doi:10.1186/s40168-015-0126-1)
Supplement: Additional file 7: Figure S3. — Microscopic observation of positive control (scrubbed human skin flakes, left), OR sample (middle), and negative control (right) with H/E staining and formalin fixation with H/E staining, formalin fixation and H/E staining (serum fixation). (PDF 9,315 kb) [file 40168_2015_126_MOESM7_ESM.pdf]

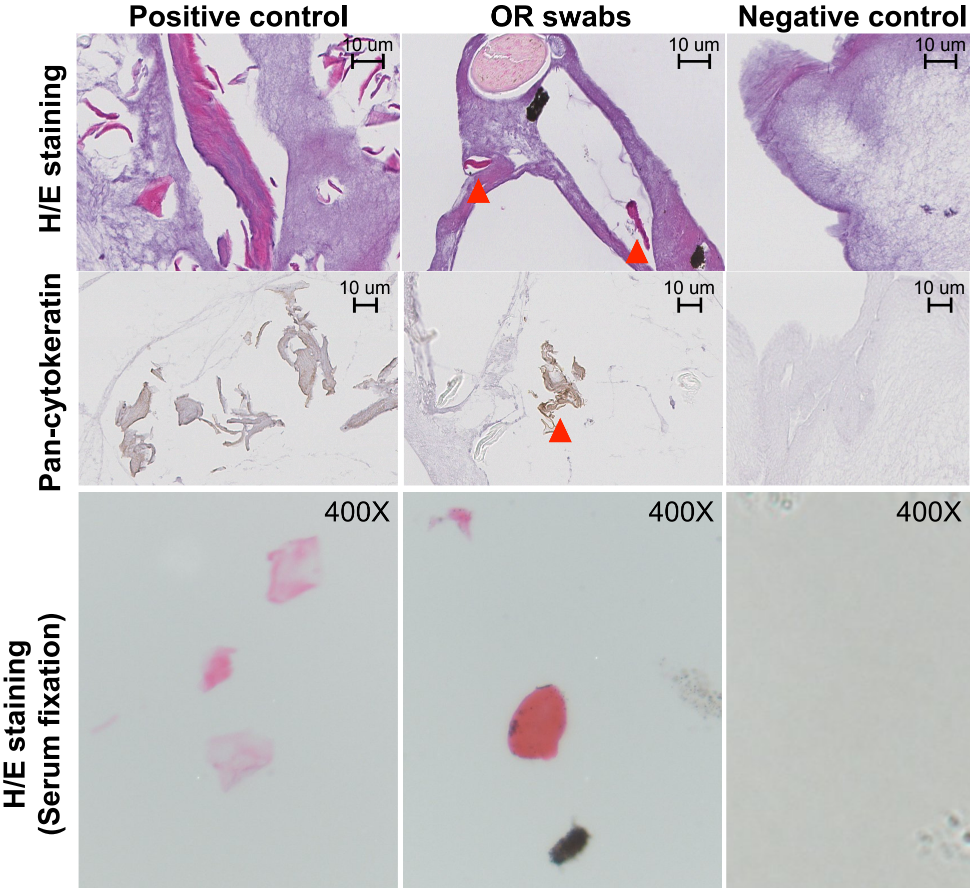
Positive control
OR swabs
Negative control
H/E staining
Pan-cytokeratin
H/E staining (Serum fixation)
10 um
400X
