# Supplementary material for: The first microbial environment of infants born by C-section: the operating room microbes
Source: Microbiome. 2015 Dec 1;3:59. doi: 10.1186/s40168-015-0126-1 (PMC4665759; doi:10.1186/s40168-015-0126-1)
Supplement: Additional file 8: Figure S4. — Source proportions for infants skin sites (foot, forehead, and volar) predicted using SourceTracker. The average contributions of human (all the mother’s sites) and operating room sources to the infant (1–7 days after birth) skin bacterial communities were predicted by SourceTracker. (PDF 109 kb) [file 40168_2015_126_MOESM8_ESM.pdf]

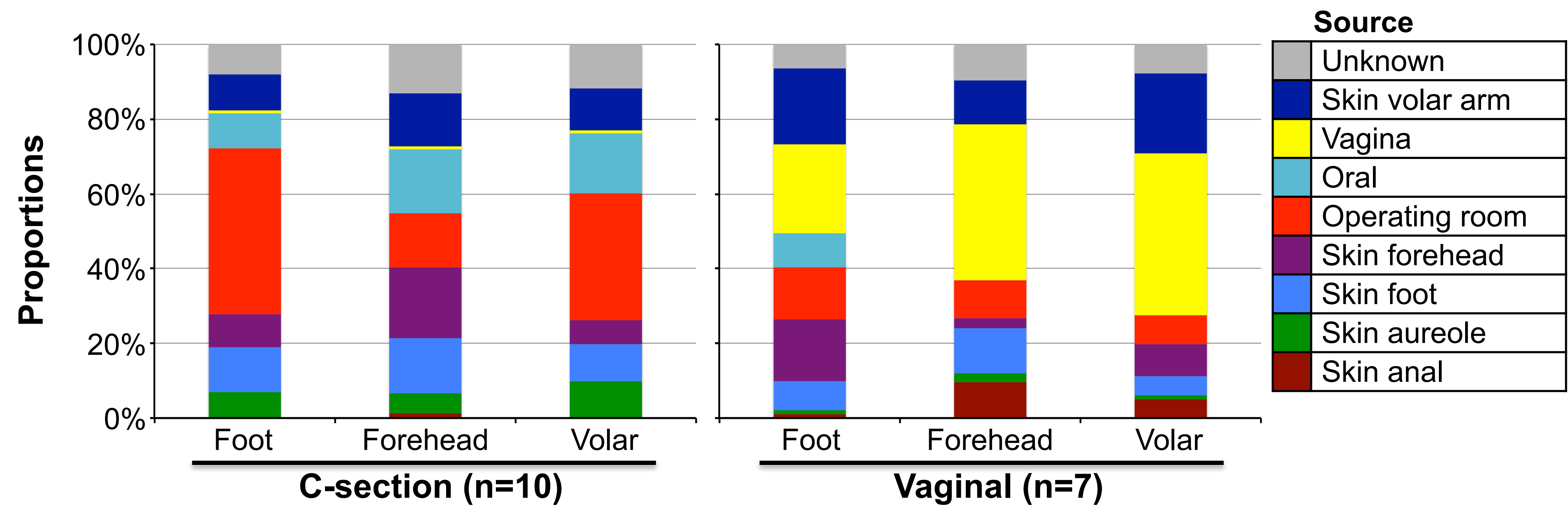

Proportions
100%
80%
60%
40%
20%
0%
Foot
Forehead
Volar
C-section (n=10)
Foot
Forehead
Volar
Vaginal (n=7)
Source
Unknown
Skin volar arm
Vagina
Oral
Operating room
Skin forehead
Skin foot
Skin aureole
Skin anal
