# Supplementary material for: The first microbial environment of infants born by C-section: the operating room microbes
Source: Microbiome. 2015 Dec 1;3:59. doi: 10.1186/s40168-015-0126-1 (PMC4665759; doi:10.1186/s40168-015-0126-1)
Supplement: Additional file 11: Figure S7. — Rarefaction plots of OR microbiota by locations using PD whole tree matrix (Left) and number of observed species (Right). All communities were rarefied at 3,194 reads. (PDF 100 kb) [file 40168_2015_126_MOESM11_ESM.pdf]

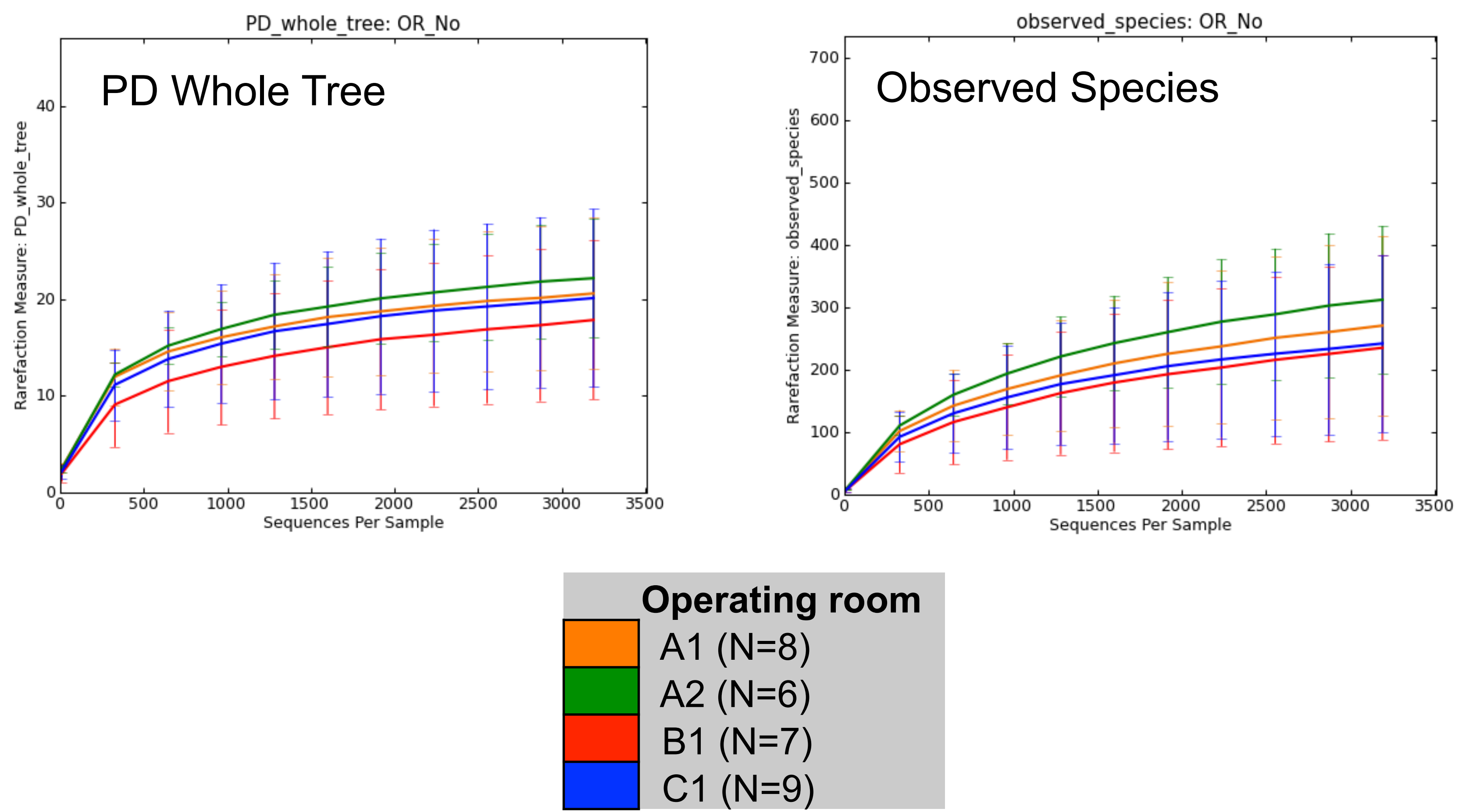

PD_whole_tree: OR_No
PD Whole Tree
Rarefaction Measure: PD_whole_tree
0
10
20
30
40
0
500
1000
1500
2000
2500
3000
3500
Sequences Per Sample
observed_species: OR_No
Observed Species
Rarefaction Measure: observed_species
0
100
200
300
400
500
600
700
0
500
1000
1500
2000
2500
3000
3500
Sequences Per Sample
Operating room
A1 (N=8)
A2 (N=6)
B1 (N=7)
C1 (N=9)
