# Supplementary material for: The first microbial environment of infants born by C-section: the operating room microbes
Source: Microbiome. 2015 Dec 1;3:59. doi: 10.1186/s40168-015-0126-1 (PMC4665759; doi:10.1186/s40168-015-0126-1)
Supplement: Additional file 13: Figure S9. — PCoA plot of bacterial communities in each OR. In upper panels, unweighted UniFrac distances were used to evaluate diversities between samples. In bottom panels, weighted UniFrac distances were used to evaluate diversities between samples. (PDF 262 kb) [file 40168_2015_126_MOESM13_ESM.pdf]

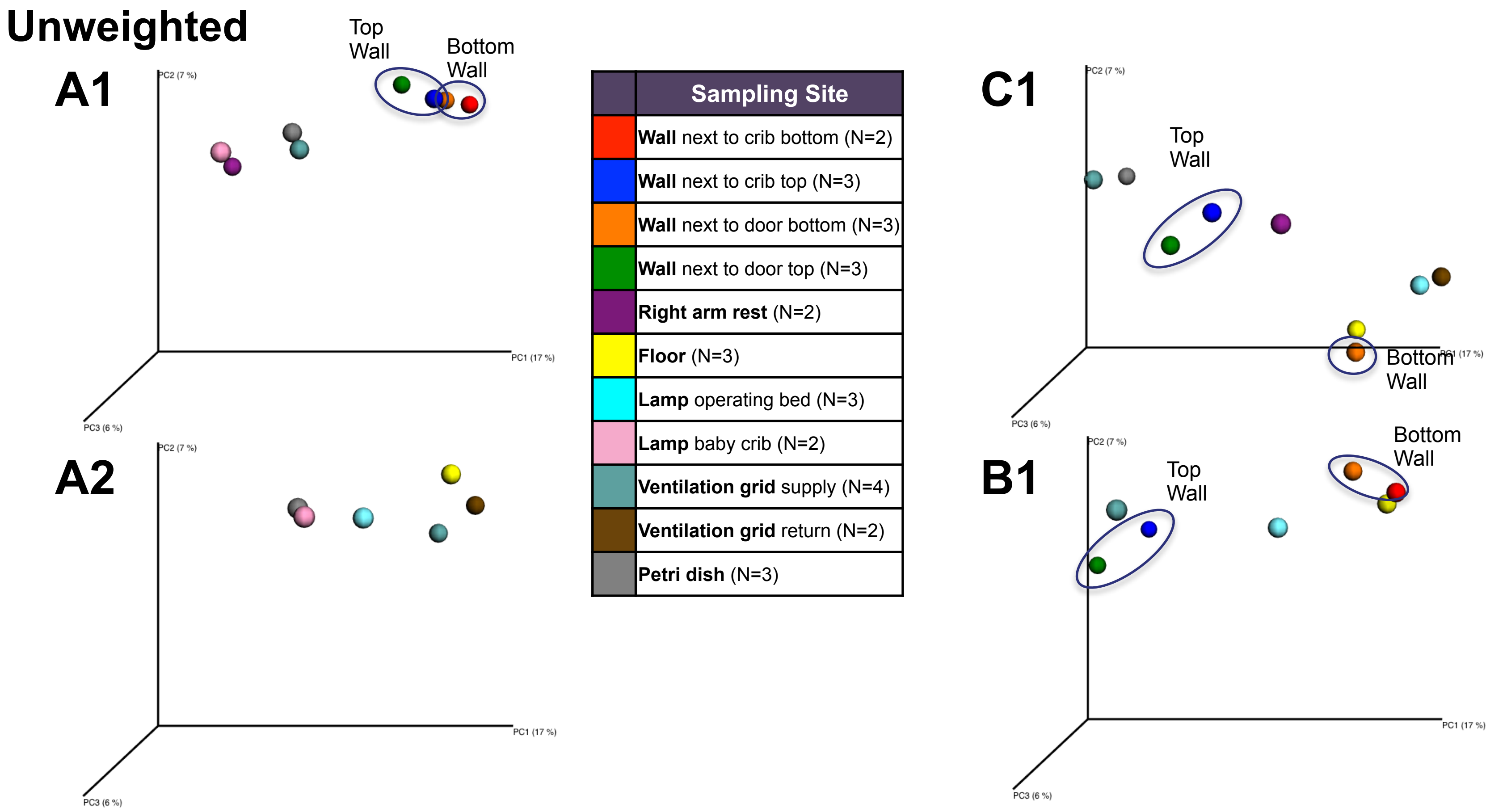
Unweighted
A1
A2
B1
C1
Top Wall
Bottom Wall
PC1 (17 %)
PC2 (7 %)
PC3 (6 %)
Sampling Site
Wall next to crib bottom (N=2)
Wall next to crib top (N=3)
Wall next to door bottom (N=3)
Wall next to door top (N=3)
Right arm rest (N=2)
Floor (N=3)
Lamp operating bed (N=3)
Lamp baby crib (N=2)
Ventilation grid supply (N=4)
Ventilation grid return (N=2)
Petri dish (N=3)

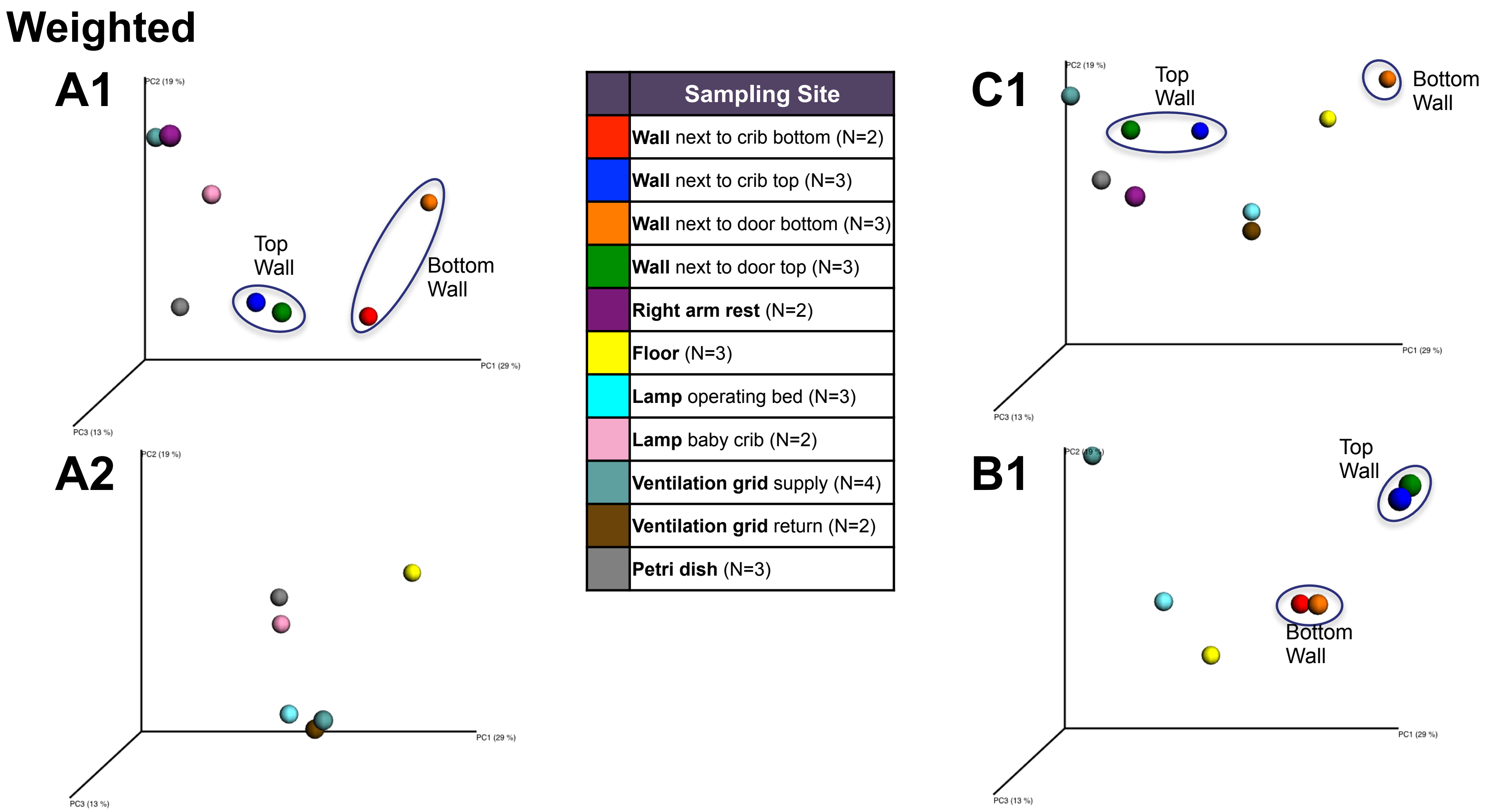
Weighted
A1
A2
B1
C1
Top Wall
Bottom Wall
PC1 (29 %)
PC2 (19 %)
PC3 (13 %)
Sampling Site
Wall next to crib bottom (N=2)
Wall next to crib top (N=3)
Wall next to door bottom (N=3)
Wall next to door top (N=3)
Right arm rest (N=2)
Floor (N=3)
Lamp operating bed (N=3)
Lamp baby crib (N=2)
Ventilation grid supply (N=4)
Ventilation grid return (N=2)
Petri dish (N=3)
